# Supplementary material for: A Genome-Wide Association Study on Liver Stiffness Changes during Hepatitis C Virus Infection Cure
Source: Diagnostics (Basel). 2021 Aug 20;11(8):1501. doi: 10.3390/diagnostics11081501 (PMC8394459; doi:10.3390/diagnostics11081501)
Supplement: Supplementary file 1 [file diagnostics-11-01501-s001.zip › Supplementary Figures_Diagnostics.pptx]

## Slide 1
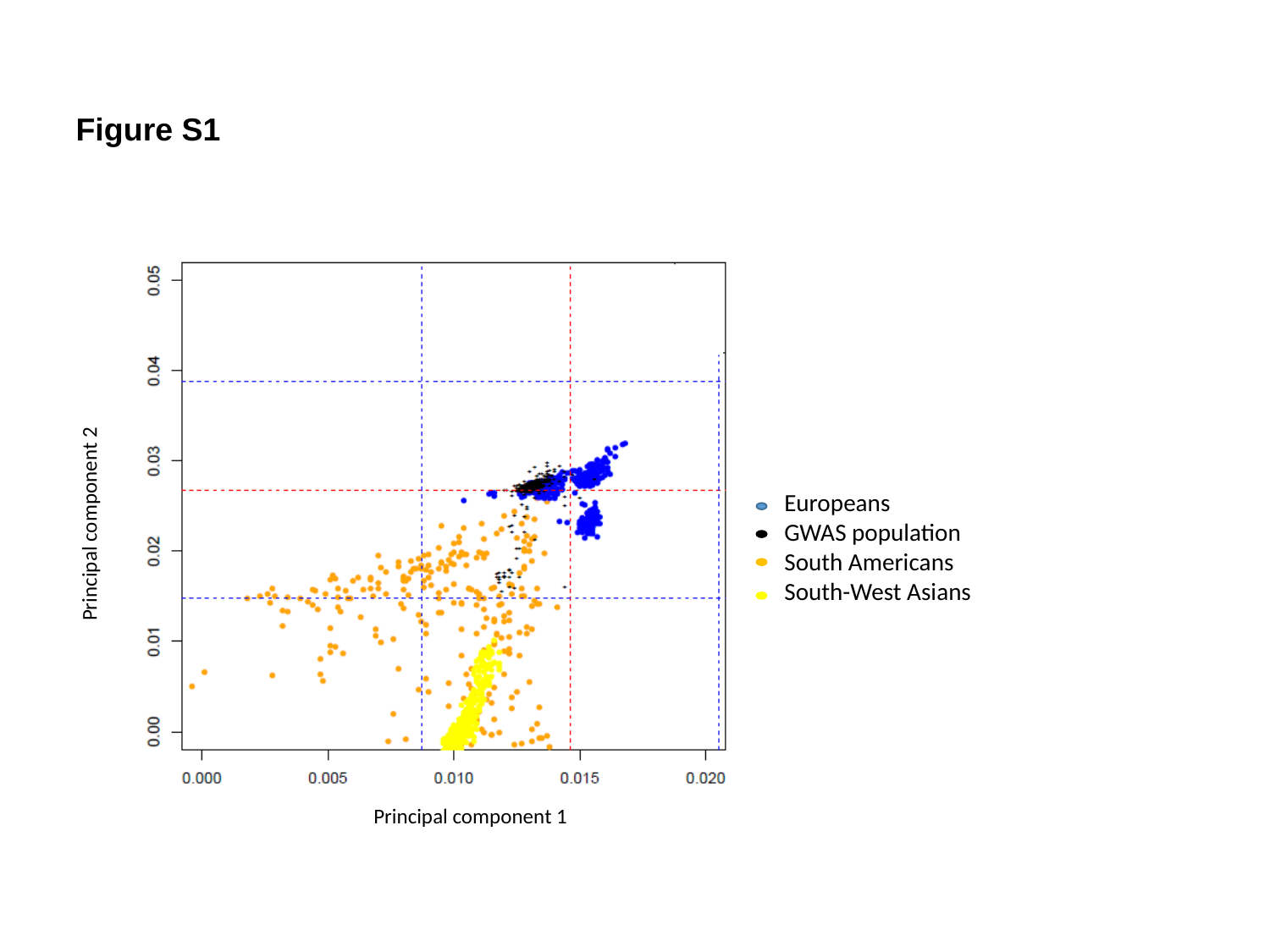

Figure S1
Principal component 2
Principal component 1
Europeans
GWAS population
South Americans
South-West Asians

## Slide 2
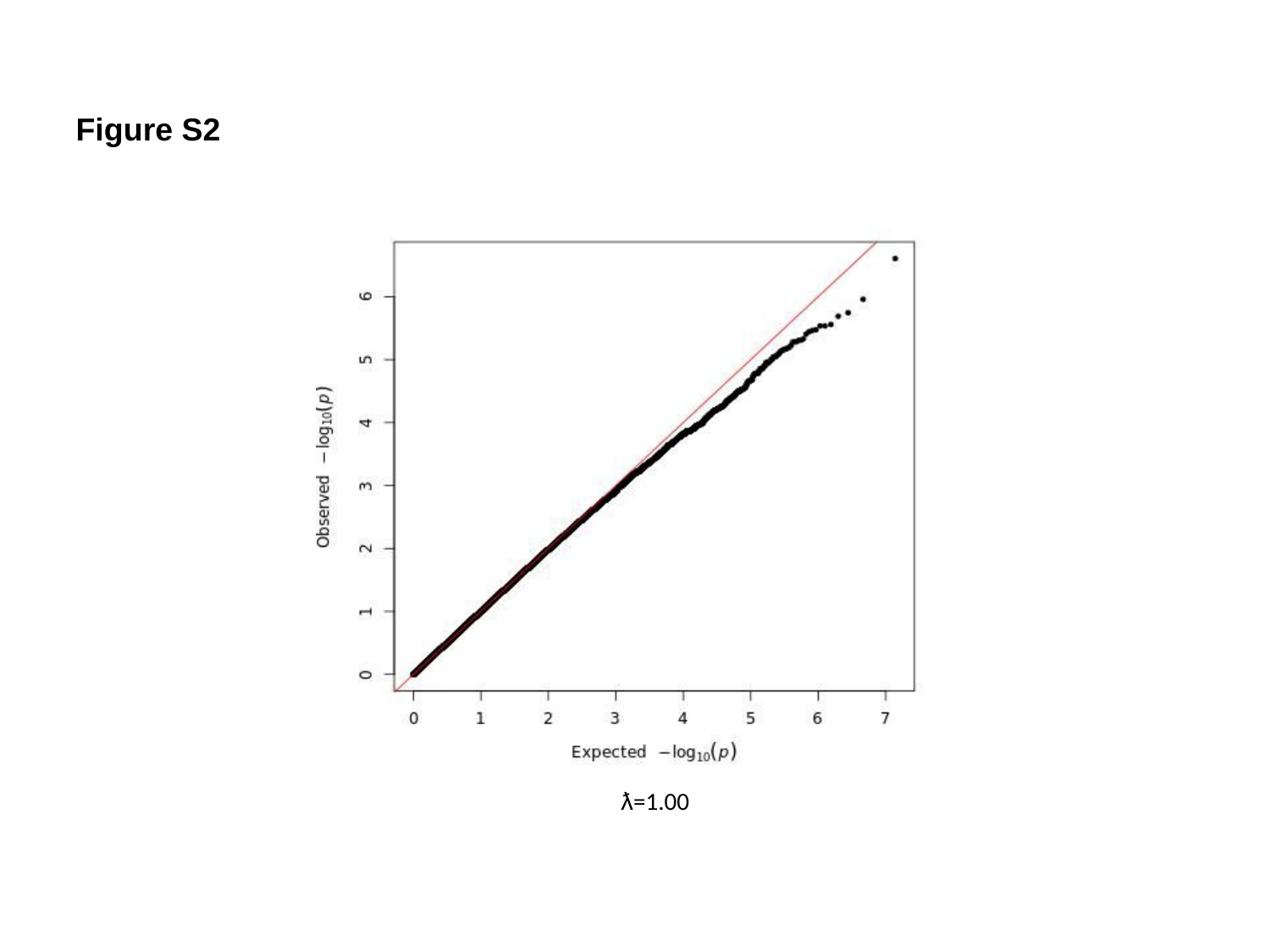

Figure S2
ƛ=1.00

## Slide 3
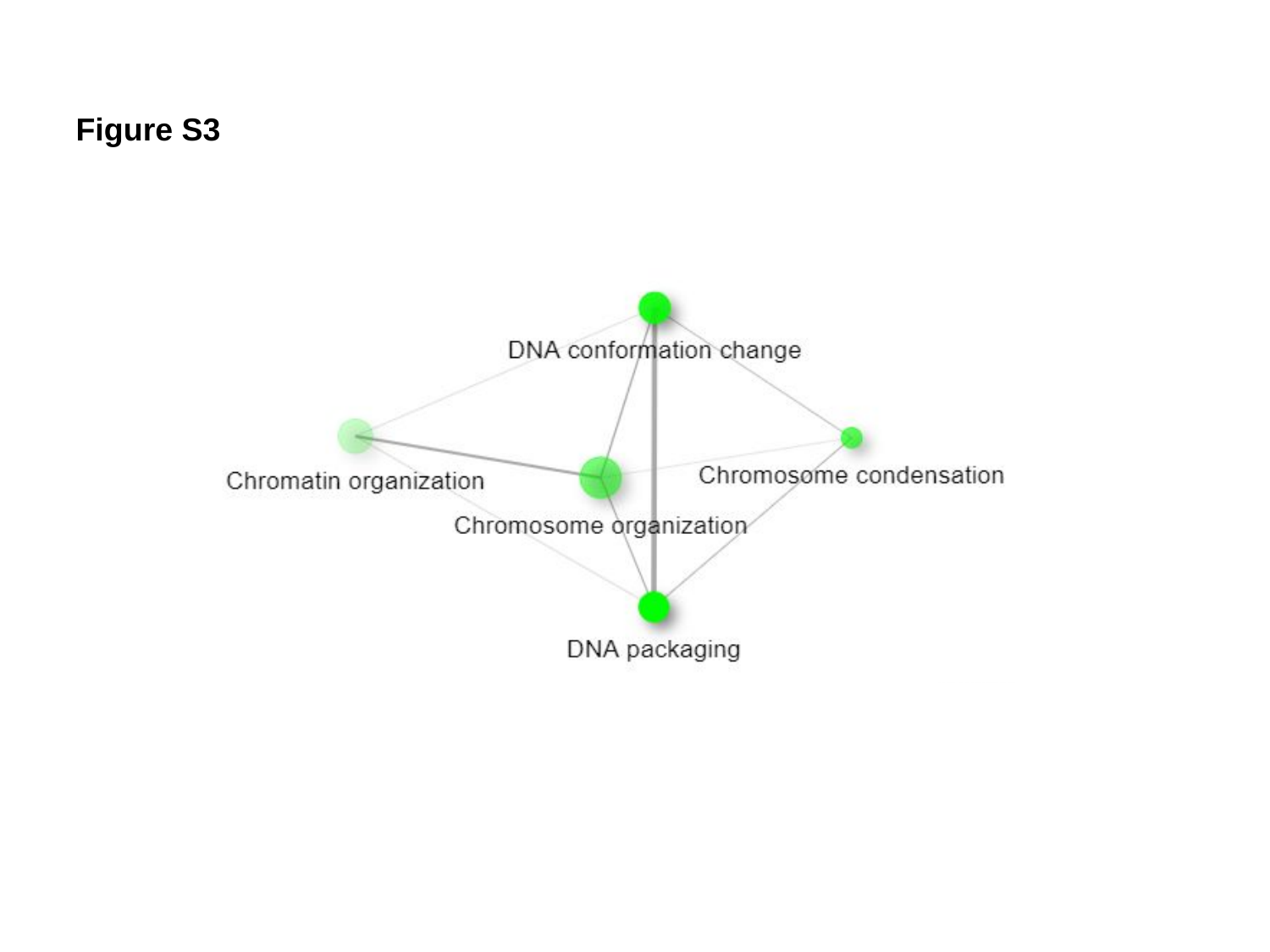

Figure S3
