# Supplementary material for: A Genome-Wide Association Study on Liver Stiffness Changes during Hepatitis C Virus Infection Cure
Source: Diagnostics (Basel). 2021 Aug 20;11(8):1501. doi: 10.3390/diagnostics11081501 (PMC8394459; doi:10.3390/diagnostics11081501)
Supplement: Supplementary file 1 [file diagnostics-11-01501-s001.zip › Supplementary tables FV_Diagnostics.pdf]

Table S1. Direct-acting antiviral regimens used in the study population for achieving sustained viral response (n=242).

DAA regimen	Treatment duration	Number (%)
IFN-based	12 weeks	8 (3.3)
	24 weeks	9 (3.7)
	48 weeks	17 (7.0)
LDV/ SOF +/- RBV	8 weeks	10 (4.0)
	12 weeks	61 (25.2)
	24 weeks	18 (7.4)
SOF /DCV +/-RBV	12 weeks	23 (9.5)
	24 weeks	15 (6.2)
SOF/SMP +/-RBV	12 weeks	14 (5.8)
	24 weeks	5 (2.0)
3D/2D +/- RBV	12 weeks	31 (12.8)
	24 weeks	5 (2.0)
SOF/VEL	12 weeks	13 (5.4)
GLE/PIB	12 weeks	1 (0.4)
EBR/GZR	12 weeks	6 (2.5)
SOF +/- RBV	24 weeks	6 (2.5)

DAA, direct-acting antiviral; IFN, interferon; LDV, ledipasvir; SOF, sofosbuvir; RBV, ribavirin; DCV, daclatasvir; SMV, simeprevir; 2D/3D, ombitasvir +/- paritaprevir +/- dasabuvir; VEL, velpatasvir; GLE, glecaprevir; PIB, pibrentasvir; GZR, grazoprevir; EBR, elbasvir

Table S2. Top 150 genes associated with the percentage of LS changes at SVR time point with respect to the LS value measured at the moment of therapy starting according to MAGMA gene-wise statistics.

CHR	START	STOP	NSNPS	NPARAM	P_SNPWISE_MEAN	GENE
1	219297192	219436207	223	18	4.07E-05	*LYPLAL1*
9	114262002	114412135	437	40	6.46E-05	*PTGR1*
14	70460934	70705787	822	55	7.10E-05	*SLC8A3*
5	126153406	126416500	645	25	0.00010801	*MARCH3*
9	114237439	114390124	410	37	0.00011144	*ZNF483*
1	20389143	20496059	261	48	0.00014326	*PLA2G2D*
14	24845738	24948731	476	37	0.00019661	*CBLN3*
14	24848882	24960548	530	32	0.0002234	*KHNYN*
14	24858972	24962042	509	32	0.00024236	*SDR39U1*
5	126328250	126459184	356	19	0.00030948	*C5orf63*
1	20336157	20468394	360	38	0.00031599	*PLA2G5*
9	114343632	114466631	406	35	0.0003228	*DNAJC25*
17	14154367	14302721	426	77	0.00044863	*HS3ST3B1*
1	75121170	75249092	338	31	0.00045145	*CRYZ*
9	114343632	114482526	472	41	0.00045816	*DNAJC25-GNG10*
1	20415823	20526879	227	56	0.00048393	*PLA2G2F*
9	21752635	21991040	477	37	0.00057078	*MTAP*
10	96393251	96545947	346	19	0.00062069	*CYP2C18*
17	58627544	58793641	76	21	0.00062748	*PPM1D*
16	53418351	53575560	302	30	0.00070929	*RBL2*
1	149862229	150032686	256	27	0.00073077	*OTUD7B*
2	48707308	48875654	544	40	0.00074046	*STON1*
14	99127950	99234103	399	43	0.00078068	*C14orf177*
2	140938996	142939270	7418	174	0.00078419	*LRP1B*
20	61924662	62059487	581	57	0.00085743	*CHRNA4*
14	24924712	25027471	434	18	0.00085913	*CMA1*
16	53475192	53588323	234	31	0.000933	*AKTIP*
6	142418372	142592085	356	24	0.00099038	*VTA1*
10	96472463	96662671	483	18	0.0010165	*CYP2C19*
16	49261828	49365742	207	32	0.0010369	*CBLN1*
5	127543601	127923735	1001	39	0.001066	*FBN2*
1	75148836	75282361	386	25	0.0011314	*TYW3*
8	15347596	15674158	1348	48	0.0011779	*TUSC3*
4	99341518	99629812	698	70	0.0012095	*TSPAN5*
6	138487123	138589627	290	20	0.0012447	*PBOV1*
14	24817992	24938494	457	43	0.0012659	*NYNRIN*
9	106806213	106953700	524	19	0.0012704	*SMC2*
8	16800334	16909674	491	45	0.0013031	*FGF20*
1	200559935	200689126	455	32	0.0013755	*DDX59*

15	88369948	88850026	1016	82	0.0013989	*NTRK3*
12	89763495	89970039	484	32	0.0015496	*POC1B*
12	13077798	13203243	312	35	0.0015641	*HEBP1*
22	30834877	30951698	410	28	0.0015919	*SEC14L4*
17	58470520	58653580	107	23	0.0016302	*APPBP2*
2	74070093	74196780	338	40	0.0016965	*ACTG2*
10	96648350	96799486	510	23	0.0019189	*CYP2C9*
16	11317141	11417452	384	28	0.0019959	*PRM3*
12	109665783	109797025	260	37	0.002055	*FOXN4*
2	48707064	49053656	1219	73	0.0021331	*STON1-GTF2A1L*
16	11319493	11420337	387	28	0.0021554	*PRM2*
12	109504222	109756031	588	51	0.0021699	*ACACB*
3	9389384	9569838	375	40	0.00221	*SETD5*
4	146490540	146631187	345	31	0.0022234	*MMAA*
5	126062315	126222712	497	41	0.0022235	*LMNB1*
16	11311714	11413160	382	30	0.0022416	*TNP2*
2	159601829	159722506	375	30	0.0023945	*DAPL1*
3	9490045	9645486	371	38	0.0024653	*LHFPL4*
9	114373851	114482526	373	33	0.0025672	*GNG10*
1	149821110	149922348	108	19	0.0026943	*BOLA1*
1	149824870	149939434	120	21	0.0027027	*SV2A*
11	117657691	117798201	375	63	0.0027503	*FXYD6*
9	109995517	110144475	487	31	0.0027675	*RAD23B*
1	149808525	149908961	104	18	0.0027975	*HIST2H2AC*
1	149806010	149908232	104	18	0.0027975	*HIST2H2BE*
1	149809019	149909466	104	18	0.0027975	*HIST2H2AB*
22	30793946	30918034	403	33	0.0028024	*SEC14L3*
1	149845209	149951449	130	17	0.0028054	*SF3B4*
1	149850543	149958791	134	16	0.0028565	*MTMR11*
11	117640790	117749408	265	52	0.0029011	*FXYD2*
6	99767279	99892082	328	24	0.0029045	*COQ3*
16	11324693	11425192	384	26	0.0029266	*PRM1*
5	6583500	6719675	529	39	0.002944	*SRD5A1*
7	133762105	133998933	521	24	0.0029505	*LRGUK*
3	195245573	195361076	336	38	0.0030623	*APOD*
5	110781731	110898234	227	36	0.003199	*STARD4*
16	11298274	11400039	366	34	0.0032347	*SOCS1*
12	27347078	27528892	519	29	0.0033337	*STK38L*
1	112888800	113053786	383	41	0.0035789	*CTTNBP2NL*
15	42817857	43063196	273	26	0.0035848	*STARD9*
12	122664111	122801068	217	27	0.0036172	*VPS33A*
21	36110098	36471595	780	102	0.0036895	*RUNX1*
2	43989611	44116039	553	36	0.0036942	*ABCG5*
20	61981561	62153993	771	62	0.0039287	*KCNQ2*
2	44016103	44155947	551	40	0.0039946	*ABCG8*
12	123187371	123305953	267	28	0.004067	*DENR*

14	92384243	92556484	498	27	0.004216	*TRIP11*
16	60342359	60443667	292	46	0.0042296	*LOC729159*
12	105674414	105815296	448	37	0.0043121	*C12orf75*
11	117721356	117850168	324	48	0.0045032	*TMPRSS13*
1	149780749	149882776	74	16	0.0046375	*HIST2H4B*
12	27074503	27217360	348	37	0.0046565	*TM7SF3*
12	27041305	27169581	290	41	0.0046793	*FGFR1OP2*
3	135634515	135916752	383	24	0.0049738	*PPP2R3A*
14	92474896	92622965	496	22	0.0049889	*ATXN3*
12	13043407	13156828	283	45	0.0052286	*GPRC5D*
15	42965760	43079417	140	18	0.0052726	*CDAN1*
2	46474541	46663842	575	67	0.0053659	*EPAS1*
6	99796534	99923263	330	25	0.0053878	*PNISR*
19	53920989	54047546	730	67	0.0054059	*ZNF813*
22	30771611	30875041	309	28	0.005436	*MTFP1*
20	37051486	37257504	331	32	0.0054567	*RALGAPB*
3	164646686	164846283	330	21	0.0054832	*SI*
3	139121726	139249662	320	16	0.0055035	*RBP2*
2	96941062	97044091	102	20	0.005626	*ITPRIPL1*
11	13249325	13458813	499	43	0.0057121	*ARNTL*
19	58587695	58716477	386	30	0.0057171	*ZNF329*
18	5839411	5945373	286	52	0.0057895	*TMEM200C*
9	99353109	99467603	96	26	0.0059402	*AAED1*
1	74983795	75189422	386	48	0.0060059	*ERICH3*
12	85380099	85708235	404	26	0.0061015	*LRRIQ1*
12	27125455	27233606	300	28	0.0062882	*MED21*
6	7540432	7662200	365	43	0.0063644	*SNRNP48*
14	54366454	54473554	246	39	0.0063662	*BMP4*
11	117640790	117797746	418	63	0.0064146	*FXYD6-FXYD2*
14	88801982	88954804	256	23	0.0064947	*SPATA7*
15	42980928	43263037	283	28	0.0065181	*TTBK2*
2	74006043	74150783	272	33	0.0065193	*STAMBP*
2	96951479	97092833	154	21	0.0067003	*NCAPH*
9	104303897	104407283	281	26	0.0067438	*PPP3R2*
13	23705060	23949304	1200	71	0.0068936	*SGCG*
11	48235413	48336330	328	17	0.0069572	*OR4X1*
1	32429295	32576460	158	28	0.0069693	*KHDRBS1*
3	136005077	136521245	713	25	0.0071261	*STAG1*
4	38615790	38753129	426	36	0.0071445	*KLF3*
1	117636209	117803582	398	45	0.0071531	*VTCN1*
19	58545204	58679793	408	29	0.0071604	*ZSCAN18*
12	32828195	32958895	453	25	0.0072134	*YARS2*
11	48277775	48378704	254	20	0.0072264	*OR4S1*
11	100850355	101050544	633	36	0.0073052	*PGR*
19	10897304	11030466	170	26	0.007326	*C19orf38*
4	76872623	76978641	339	18	0.0073277	*CXCL9*

1	16334264	16450127	508	39	0.0073361	*FAM131C*
11	48296493	48397482	202	19	0.0073388	*OR4C3*
9	104246131	104375626	320	29	0.0073757	*RNF20*
1	32662818	32764461	112	23	0.0074535	*FAM167B*
3	135817760	135965522	145	21	0.0074553	*MSL2*
2	38843052	39011909	384	41	0.0074621	*GALM*
1	149774181	149874687	66	13	0.0075391	*HIST2H3A*
2	220442292	220556702	135	33	0.007568	*SLC4A3*
2	121443441	121800229	885	81	0.007618	*GLI2*
5	134620071	134785645	291	43	0.0078287	*H2AFY*
22	30742930	30871291	385	31	0.0078979	*SEC14L2*
8	30446117	30553473	197	25	0.0080259	*SMIM18*
5	6549352	6683473	615	45	0.0080348	*NSUN2*
1	149772628	149873161	64	13	0.0080366	*HIST2H2AA4*
3	57211765	57357499	226	22	0.0080378	*APPL1*
4	76892269	76994689	389	18	0.0080749	*CXCL10*
3	57181866	57311656	190	21	0.0080785	*HESX1*
9	98947588	99114662	490	47	0.008083	*HSD17B3*
12	10095660	10203381	378	33	0.0080938	*CLEC1B*

CHR, Chromosome; BP, base pair position according to UCSC genome browser (NCBI37/hg19) and dbSNP build 150; NSNPS, number of SNPs in the gene region; NPARAM, number of SNPs used in the analysis.
